# Forty-Eight-Year Functionality of Björk-Shiley Aortic and Mitral Valves in One Patient

**DOI:** 10.7759/cureus.44976

**Published:** 2023-09-10

**Authors:** Taylor McManus, Philip D Zinn

**Affiliations:** 1 Cardiology, University of Texas Medical Branch, Galveston, USA; 2 Cardiology, Cardiology Clinic of San Antonio, San Antonio, USA

**Keywords:** cardiac surgery, rheumatic fever, valve replacement, aortic valve, mitral valve

## Abstract

The Björk-Shiley mechanical valve was the first tilting-disc valve used to replace aortic and mitral valves. We present a case of a double aortic and mitral valve replacement with both valves functioning properly over 48 years after the original implantation. Minimal negative changes are evident in either valve despite the progression of cardiac abnormalities, including the development of a dilated cardiomyopathy associated with chronic systolic and diastolic heart failure and the implantation of an implantable cardioverter defibrillator (ICD). Our patient's case appears to be the most significant example of double-valve replacement longevity described in the literature.

## Introduction

The Björk-Shiley valve was initially introduced for clinical use in January 1969 and used until the 1980s when a structural change in the valve caused increasing levels of adverse effects, including thrombosis and blockage of leaflets [1]. Several adjustments to the device were made over its lifespan, beginning with a Delrin disc, Pyrolyte carbon disc, convex-concave valve with openings of 60 degrees, then 70 degrees, and most recently, the Monostrut valve [2]. The risk of some complications was significantly greater in patients with double valve replacements, notably anticoagulant-induced hemorrhages, endocarditis, and overall morbidity and mortality [3].

## Case presentation

**Figure 1:**
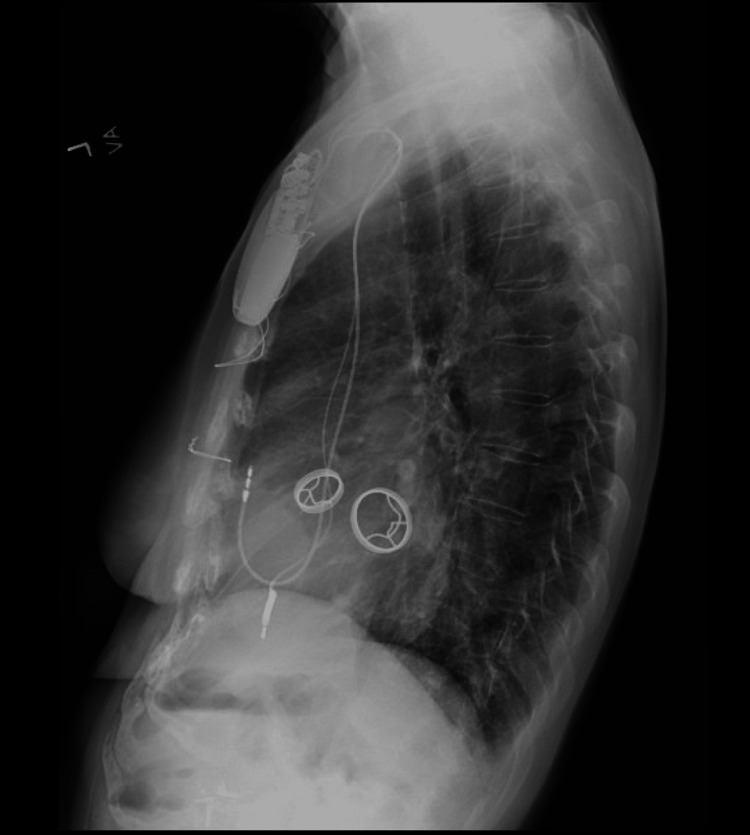
Lateral view of intact mitral and aortic valves (January 2023)

Our patient had a history of rheumatic fever with presumably rheumatic valvular disease and underwent a double valve replacement on December 3rd, 1974, at age 18, following septicemia from a complicated at-home childbirth. The valves implanted were a Björk-Shiley pyrolite carbon 23-mm aortic valve (serial #23ABP4533) and a 29-mm mitral valve (serial #23MBRP3500) (Figure 1). Post-operatively, her left ventricular size and function were normal, and she was placed on digoxin and long-term anticoagulation with warfarin. The patient had initially had difficulties managing warfarin anticoagulant therapy and was found to have an anti-cardiolipin antibody. Clinical findings of both valve replacements have been normal for the entirety of the life of the prostheses. She has had numerous echocardiograms between 1994 and 2023, and a summary of the left ventricular ejection fractions is listed in Table 1. 

**Table 1:** Ejection fraction tracking between the years 1994-2023

Date	Ejection fraction
June 1994	74%
January 1999	72%
April 2006	55-60%
May 2012	55-60%
August 2017	35-40%
January 2021	25%
January 2023	22%

**Figure 2:**
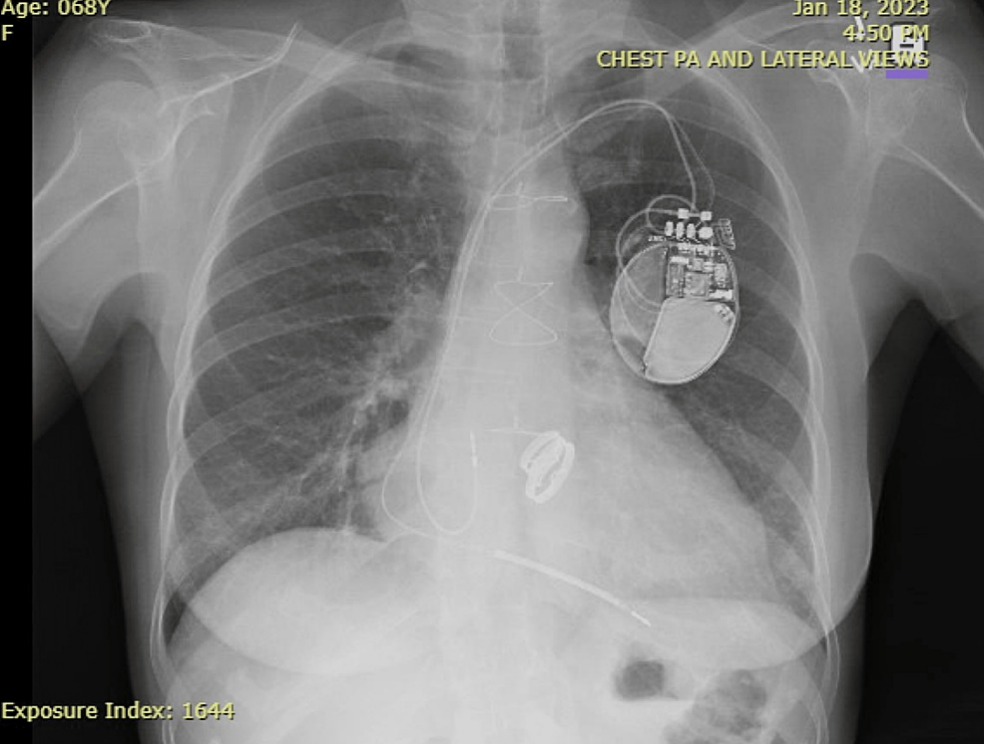
Posterior anterior view (January 2023)

In 2014, she developed congestive heart failure, and an echocardiogram revealed a decrease in her left ventricular function with a left ventricular ejection fraction (LVEF) of 30-35% with apical, anteroapical and apical septal akinesis. She had a cardiac computed tomography angiography (CTA), which showed her coronary arteries were normal, but she had an apical aneurysm consistent with findings on her echocardiogram and a prior apical myocardial infarction. Her congestive heart failure was treated, and she subsequently had a dual chamber pacemaker defibrillator (DDD/ICD) implanted in 2014 (Figure 2). We hypothesize that she may have had an embolus to her left anterior descending (LAD) coronary artery, resulting in what appears to be a myocardial infarction in the setting of normal coronary arteries. Her most recent echocardiogram from 2023 revealed normally functioning, well-seated aortic and mitral valve prostheses. Her LVEF has decreased to 20-25% despite medical therapy. She has symptomatic chronic systolic and diastolic congestive heart failure; however, both valves continue to function normally. On the most recent echocardiogram, the prosthetic mitral valve had an effective orifice area (EOA) of 3.1 cm^2^ with an indexed effective orifice area (EOAi) of 1.93 cm^2^/m^2^, and the prosthetic aortic valve had an EOA of 1.7 cm^2^ and an EOAi of 1.06 cm^2^/m^2^. Her medical history also includes hypertension, hyperlipidemia, paroxysmal atrial fibrillation, and nonsustained ventricular tachycardia.

In 2022, she had to undergo extensive foot surgery, and her warfarin was temporarily stopped. Despite bridging anticoagulation with enoxaparin, she suffered a transient ischemic attack (TIA), but fortunately, there was no evidence of any long-term neurologic sequelae. 

## Discussion

The current trend in valve replacement has shifted toward using more bioprosthetic valves, with a marked increase in the use of transcatheter aortic valve replacement (TAVR), particularly in older individuals. For individuals less than 50 years old, the current American College of Cardiology and the American Heart Association guidelines (ACC/AHA) recommend a mechanical valve for aortic valve replacement. However, many of these patients can avoid a prosthetic valve using a pulmonic autograph in the aortic position (the Ross procedure). In patients undergoing mitral valve replacement, current ACC/AHA guidelines recommend a mechanical valve under 65 years old [4]. These guidelines assume mechanical valves will have significantly greater longevity than bioprosthetic valves; thus, it is essential to know that patients can potentially survive many decades with a mechanical valve. The Björk-Shiley valve was used extensively in the 70s and 80s as a prosthetic valve replacement. Researchers have described the decades of longevity seen in Björk-Shiley single valve replacements. Nitter-Hauge et al. reported a series of 25 patients who had double aortic and mitral valve replacement with Björk-Shiley tilting disc valves (pyrolite) [5]. As recently as 2015, Soofi et al. had the longest follow-up for a Björk-Shiley valve replacement patient at 42 years [6]. However, our patient has had two functioning replacement valves, mitral and aortic, for over 48 years. To our knowledge, this case would represent the longest reported longevity of a Björk-Shiley double-valve replacement. The longevity of older mechanical valves should serve to reassure both patients and physicians that these valves are quite durable and can last for many decades.

## Conclusions

Overall, the Björk-Shiley valves have held up quite well, clinically maintaining normal function. We report a case of a 68-year-old patient with a double-valve Björk-Shiley pyrolite carbon disc replacement with over 48 years of function. Our case shows the necessity for long-term anticoagulation and proactive verification of the stability of any mechanical valve using lifelong imaging studies and follow-up. Advancements in valve replacement surgery do not make these cases obsolete but should instead add value to the success of decades worth of use.
